# Epidemiological and clinical characteristics of COVID-19 reinfection during the epidemic period in Yangzhou city, Jiangsu province

**DOI:** 10.3389/fpubh.2023.1256768

**Published:** 2023-09-13

**Authors:** Yin Wang, Jie Liang, Huimin Yang, Liguo Zhu, Jianli Hu, Lishun Xiao, Yao Huang, Yuying Dong, Cheng Wu, Jun Zhang, Xin Zhou

**Affiliations:** ^1^Yangzhou Center for Disease Control and Prevention, Yangzhou, Jiangsu, China; ^2^Jiangsu Provincial Center for Disease Control and Prevention, Nanjing, Jiangsu, China; ^3^Department of Biostatistics, School of Public Health, Xuzhou Medical University, Xuzhou, Jiangsu, China

**Keywords:** SARS-CoV-2, reinfection, epidemiological characteristics, clinical characteristics, epidemic

## Abstract

**Background:**

With the continuous progress of the epidemic of coronavirus disease 2019 (COVID-19) infection and the constant mutation of the virus strain, reinfection occurred in previously infected individuals and caused waves of the epidemic in many countries. Therefore, we aimed to explore the characteristics of COVID-19 reinfection during the epidemic period in Yangzhou and provide a scientific basis for assessing the COVID-19 situation and optimizing the allocation of medical resources.

**Methods:**

We chose previously infected individuals of severe acute respiratory syndrome coronavirus 2 (SARS-CoV-2) reported locally in Yangzhou city from January 2020 to November 30, 2022. A telephone follow-up of cases was conducted from February to March 2023 to collect the COVID-19 reinfection information. We conducted a face-to-face survey on that who met the definition of reinfection to collect information on clinical symptoms, vaccination status of COVID-19, and so on. Data were analyzed using SPSS 19.0.

**Results:**

Among the 999 eligible respondents (92.24% of all the participants), consisting of 42.28% males and 57.72% females, the reinfection incidence of females was significantly higher than that of male cases (χ^2^ = 5.197, *P* < 0.05); the ages of the respondents ranged from 1 to 91 years, with the mean age of 42.28 (standard deviation 22.73) years; the most of the sufferers were infected initially with Delta variant (56.88%), followed by the Omicron subvariants BA.1/BA.2 (39.52%). Among all the eligible respondents, 126 (12.61%) reported COVID-19 reinfection appearing during the epidemic period, and the intervals between infections were from 73 to 1,082 days. The earlier the initial infection occurred, the higher the reinfection incidence and the reinfection incidence was significantly increased when the interval was beyond 1 year (*P* < 0.01) .119 reinfection cases (94.4%) were symptomatic when the most common symptoms included fever (65.54%) and cough (61.34%); compared with the initial infection cases, the proportion of clinical symptoms in the reinfected cases was significantly higher (*P* < 0.01). The reinfection incidence of COVID-19 vaccination groups with different doses was statistically significant (*P* < 0.01). Fewer reinfections were observed among the respondents with three doses of COVID-19 vaccination compared to the respondents with two doses (χ^2^ = 14.595, *P* < 0.001) or without COVID-19 vaccination (χ^2^ =4.263, *P* = 0.039).

**Conclusion:**

After the epidemic period of COVID-19, the reinfection incidence varied with different types of SARS-CoV-2 strains. The reinfection incidence was influenced by various factors such as virus characteristics, vaccination, epidemic prevention policies, and individual variations. As the SARS-CoV-2 continues to mutate, vaccination and appropriate personal protection have practical significance in reducing the risk of reinfection.

## 1. Introduction

Reinfections might occur with many respiratory viruses, including human coronaviruses, mainly due to weak or waning initial immune response, reinfection with another genotype/species or the high variability of the viruses (1). Reinfection was defined as recovery followed by a new infection due to the same variant previously infected or a new variant of the agent (2). In August 2020, To et al. (3) first described an asymptomatic patient from Hong Kong with a positive SARS-CoV-2 PCR test from a sample collected 142 days after a first symptomatic COVID-19 episode. Since then, the reinfection of COVID-19 in previously infected individuals has been reported in many parts of the world (4, 5), with the reinfection incidence of different variants varying considerably in different periods and regions. Due to the influence of epidemic prevention policies and the characteristics of the SARS-CoV-2, the reinfection incidence of COVID-19 in previously infected individuals was low before the emergence of the Omicron variant. Up to March 2021, a meta-analysis indicated the overall prevalence of reinfection among COVID-19 patients was 3‰ (95% confidence interval [CI]:0.8–5‰) (6). Since the worldwide outbreak of the Omicron variant in November 2021, the reinfection incidences reported in multiple regions have significantly increased. According to an analysis of a survey conducted in the UK, the highest reinfection incidence among individuals previously infected with different strains from February 2020 to November 2022 could reach 16.6% (7). The SARS-CoV-2 Omicron variant spread rapidly nationwide since November 2022, seriously affecting the health of residents. In previous studies, the factors associated with the infection rate of COVID-19 might include socio-demographic factors such as gender, age, and occupation, but the results were not entirely consistent (8, 9), whereas vaccination has presented a certain protective effect against COVID-19 (10, 11). And in the few studies of reinfection in China, the factors influencing reinfection rates were similar to those of initial infection (12, 13). This study was based on the survey of COVID-19-infected individuals in Yangzhou City during the epidemic period from January 2020 to November 30, 2022, to explore the characteristics of COVID-19-reinfection and the influencing factors, and provide a scientific basis for assessing the COVID-19 situation and optimizing the allocation of medical resources.

## 2. Participants and methods

### 2.1. Participants

Following the China Information System for Disease Control and Prevention (CISDCP), COVID-19 previously infected individuals reported locally in Yangzhou city from January 2020 to November 30, 2022, were chosen.

### 2.2. Methods

#### 2.2.1. Investigation methods

Trained and assessed epidemiological investigators conducted the first round of telephone follow-up surveys on the cases or their guardians included in this study, followed by the second round of face-to-face surveys on the one that met the definition of reinfection.

#### 2.2.2. Investigation content

The telephone survey mainly included demography basic information, reinfection status, SARS-CoV-2 nucleic acid or antigen test results, and information about close contacts, whether the contacts had positive antigen tests or COVID-19 symptoms.

Face-to-face surveys were conducted to collect information on the first infection of COVID-19, the diagnosis and treatment of the latest infection of COVID-19, and so on. The COVID-19 vaccination information was obtained from the records of the Jiangsu Province Vaccination Integrated Service Management Information System.

#### 2.2.3. Definition of reinfection

(1) Confirmed case of SARS-CoV-2 reinfection: Identified as those who tested positive for SARS-CoV-2 nucleic acid or antigen more than 60 days after the last positive SARS-CoV-2 nucleic acid or antigen irrespective of symptoms.
(2) Probable case of SARS-CoV-2 reinfection: Defined as those who met an acute onset or worsening of any following signs or symptoms (fever, fatigue, cough, sore throat, hyposmia/anosmia, nasal congestion, runny nose, conjunctivitis, myalgia, diarrhea, etc.), and had an epidemiological history (close contact with the case of positive SARS-CoV-2 nucleic acid test/rapid antigen test for COVID-19 or of similar symptoms), and the interval from the last positive SARS-CoV-2 nucleic acid test exceeded more than 60 days.

#### 2.2.4. Genome sequencing

During January 2020 and November 30, 2022, nasopharyngeal swab samples from COVID-19-infected individuals reported locally in Yangzhou city were collected by the CDCs along with designated hospitals for COVID-19 diagnosis and treatment. These samples were promptly dispatched to the Yangzhou CDC for further analysis, including nucleic acid testing and genome sequencing.

Genome sequencing was implemented on all SARS-CoV-2 nucleic acid test-positive cases with cycle threshold (Ct) values ≤32. Strains of the local case reported in 2020 were all original. The chain of transmission for local outbreaks of COVID-19 in both 2021 July-August and 2022 was explicit, and the strains were Delta variant and Omicron subvariants BA.5.2/BF.7, respectively.

#### 2.2.5. Statistical analysis

Epidemiological investigators conducted telephone and face-to-face questionnaires. Data were entered and managed using EpiData 3.1 and analyzed using SPSS 19.0. Categorical variables were compared using the Chi-squared test or Fisher's exact test. Whereas, the Shapiro-Wilk test was used to assess the normality of continuous variables which were expressed by mean±standard ($\bar{x}\pm s$) deviation after conforming to normal distribution. Homogeneity of variance between the groups was checked by Levene's test and the data were analyzed by *t*-test when homogeneity of variance. Multivariable logistic regression was done and a *p*-value of ≤ 0.05 was used to declare the level of significance.

#### 2.2.6. Quality control

The survey group consisted of an investigation-leading group, a technical guidance group, and a quality control group, and epidemiological investigators were trained and assessed. The survey was conducted strictly under unified investigation methods and questionnaires. The investigators checked and corrected the questionnaire to ensure that there were no missing items or logic errors after the everyday survey. 5–10% of the questionnaire were randomly selected for review.

## 3. Results

### 3.1. Basic characteristics of respondents

Between January 2020 and November 30, 2022, a total of 1,083 previously infected individuals were reported locally, of which 999 completed telephone surveys with a response rate of 92.24%. The main period for the initial positive SARS-CoV-2 nucleic acid test of all previously infected individuals was July 28 to August 26, 2021 (*n* = 570, 52.63%). 42.28% of the respondents were male and 57.72% were female, with a sex ratio (M/F) of 0.73:1. The ages of the respondents ranged from 1 to 91 years old, with a mean age of 42.28 (standard deviation 22.73) years. Most of the sufferers were infected initially with the Delta variant (56.88%), followed by the Omicron subvariants BA.1/BA.2 (39.52%) (Table 1).

**Table 1:** Types of SARS-CoV-2 strains of previously infected individuals during the epidemic period in Yangzhou, China.

**Virus type**	**Number of infection cases (*N =* 1,083)**	**Number of respondents (*N =* 999)**	**Response rate (%)**
Delta variant (%)	616 (56.88)	553 (55.36)	89.77
Omicron variant (%)	428 (39.52)	408 (40.84)	95.33
Original strai*n* (%)	39 (3.60)	38 ( 3.80)	97.44

### 3.2. Interval of reinfection

**Figure 1:**
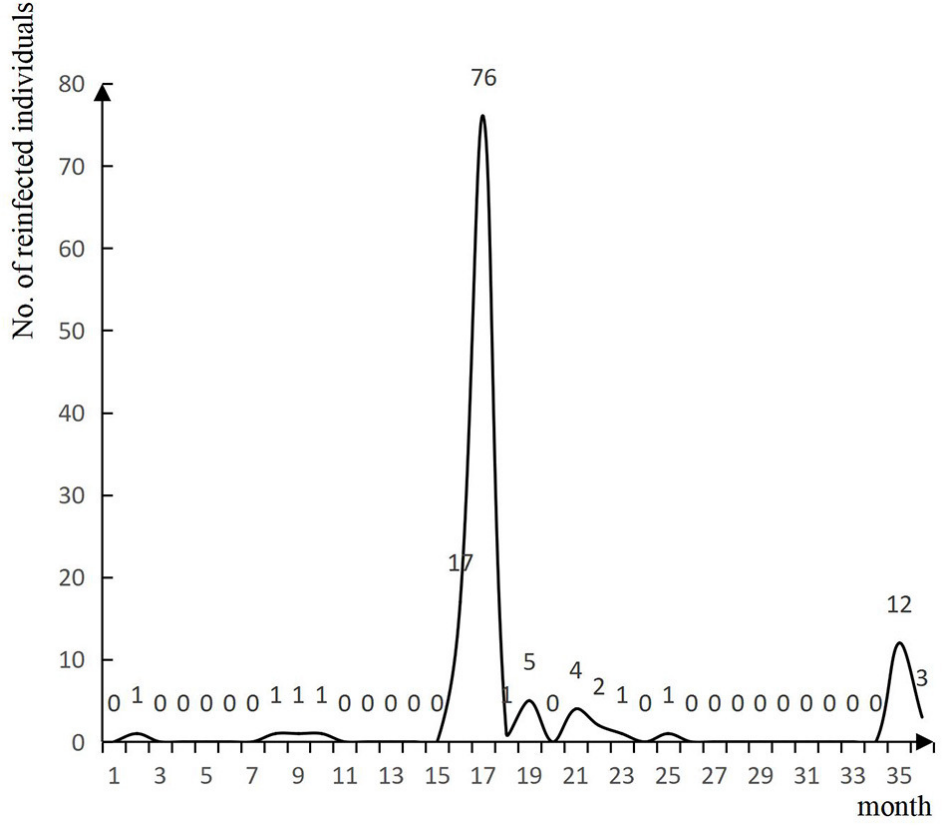
Intervals between infections in previously infected individuals.

No individuals previously infected with COVID-19 were found to be reinfected before November 2022. Among all the 999 eligible respondents reported between November 2022 and January 2023, 126 met the definition of reinfection, with a reinfection incidence of 12.61%. Whereas, the intervals between infections ranged from 73 to 1,082 days, and the median interval (P25–P75) was 508 days (498–530) (Figure 1).

### 3.3. Epidemiological characteristics of reinfected individuals

There was no statistically significant difference in reinfection incidences among different occupations, however, the statistical difference was observed in the following factors between the two groups, gender, age, interval from initial infection to the investigation, and type of SARS-CoV-2 strains. The results showed that the reinfection incidence of females was significantly higher than that of male cases (χ^2^=5.197, *P* < 0.05). The difference in the average age between reinfected and non-reinfected groups was statistically significant (*P* < 0.05). The earlier the initial infection occurred, the higher the reinfection incidence and the reinfection incidence was significantly increased when the interval was beyond 1 year (*P* < 0.01). Considering the impact of observation time on the reinfection incidence of different strains, the reinfection status of different SARS-CoV-2 strains was analyzed using incidence density. The incidence densities of the original strain, Delta variant, and Omicron variant were 168.42, 127.56, and 19.70 per 1,000 person-years, respectively (Tables 2, 3).

**Table 2:** Reinfection incidences by gender, age, occupation, interval from initial infection to investigation and type of SARS-CoV-2 strains.

**Variables**	**Reinfection status**		**Reinfection incidence (%)**	**χ^2^/F/t**	**P-value**
	**Reinfected (*****N** =* **126)**	**Non reinfected (*****N** =* **873)**			
**Gender**, ***n*** **(%)**				5.197	0.023
Male	53 (42.06)	462 (52.92)	10.30		
Female	73 (57.94)	411 (47.08)	15.10		
Mean age ± SD, yr	42.65 ± 20.39	46.44 ± 19.73	13.01	−2.031	0.043
**Occupation**, ***n*** **(%)**				0.509	0.775
Student and preschooler	15 (11.90)	96 (11.00)	13.50		
Medical staff	3 (2.38)	14 (1.60)	17.60		
Others	108 (85.71)	763 (87.40)	12.40		
**Interval from initial infection to investigation**, ***n*** **(%)**				84.950	< 0.001
< 6 months	1 (0.79)	346 (39.63)	0.30		
6 months to 1 year	3 (2.38)	56 (6.41)	5.10		
>1 year	122 (96.83)	471 (53.95)	20.60		
**Type of SARS-CoV-2 strains**, ***n*** **(%)**				66.489	< 0.001
Omicron variant	4 (3.17)	404 (46.28)	1.00		
Delta variant	106 (84.13)	447 (51.20)	19.17		
original strain	16 (12.70)	22 (2.52)	42.11		

**Table 3:** The relative risk and results of the logistic regression model among previously infected individuals.

**Variables**	**OR (95%CI)**	**P-value**
**Gender**		
Male	1	
Female	1.578 (1.061–2.259)	0.023
Age, yr	0.994 (0.985–1.003)	0.203
**Interval from initial infection to investigation**		
< 6 months	1	
6 months to 1 year	89.813 (12.489–645.870)	< 0.001
>1 year	18.211 (1.862–178.118)	0.013
**Type of SARS-CoV-2 strains**		
original strain	1	
Omicron variant	0.325 (0.165–0.641)	0.001
Delta variant	0.014 (0.004–0.044)	< 0.001

### 3.4. Clinical characteristics of reinfected individuals

Among the 126 reinfected individuals, 119 (94.4%) were symptomatic. The most common symptoms included fever (*n* = 78,65.54%), cough (*n* = 73,61.34%), sore throat (*n* = 22,18.49%), feebleness (*n* = 19,15.97%), headache (*n* = 13,10.92%), hyposmia/anosmia (*n* = 6,5.04%) and symptoms of gastrointestinal (*n* = 3,2.52%). Additionally, 121 (96.03%) took medication on their own, and only the remaining 5 (3.97%) sought medical attention. There were no critical or hospitalized cases. Compared to the initial infection, the proportion of clinical symptoms in the reinfected population was significantly higher (*P* < 0.01). The proportion of clinical symptoms such as fever, cough, sore throat, and fatigue among the symptomatic individuals was not statistically significant at the time of initial infection and reinfection (Table 4).

**Table 4:** Clinical characteristics of reinfected individuals.

**Variables**	**Infection (*N =* 126)**	**Reinfection (*N =* 126)**	**χ^2^**	**P-value**
**Whether symptomatic**			27.232	< 0.001
Yes	87 (69.05)	119 (94.44)		
No	39 (30.95)	7 (5.56)		
**Symptom**				
Fever	66 (75.86)	78 (65.54)	2.542	0.111
Cough	48 (55.17)	73 (61.34)	0.790	0.374
Sore throat	17 (19.54)	22 (18.49)	0.036	0.849
Fatigue	9 (10.34)	19 (15.97)	1.352	0.245
**Fever** ^*^			4.640	0.098
Low-grade	14 (21.21)	9 (11.54)		
Moderate-grade	27 (40.91)	45 (57.69)		
High-grade	25 (37.88)	24 (30.77)		

^*^Low-grade:37.3°C−37.9°C; Moderate-grade: 38°C−38.9°C; High-grade:39°C and above.

### 3.5. Vaccination status of reinfected individuals

17.32% of the reinfected individuals received three or more doses of COVID-19 vaccination, and 14.91% received two doses. The reinfection incidence of COVID-19 vaccination groups with different doses was statistically significant (*P* < 0.01), fewer reinfections were observed among the respondents with three doses of COVID-19 vaccination compared to the respondents with two doses (χ^2^=14.595, *P* < 0.001) or without COVID-19 vaccination (χ^2^=4.263, *P* = 0.039). However, there was no statistically significant difference in the reinfection incidence between those who received COVID-19 vaccination or not in the past six months or after the initial infection (Table 5).

**Table 5:** Vaccination status of COVID-19 among reinfected individuals.

Vaccination status^*^	**Reinfection status**		**reinfection incidence (%)**	**χ^2^**	**P-value**

	**Reinfected (*****N** =* **126)**	**Non reinfected (*****N** =* **873)**			
**Vaccination doses**				15.190	0.002
Unvaccinated	19	111	14.60		
1 dose	9	49	15.50		
2 doses	56	264	17.50		
≥3 doses	42	449	8.60		
**Vaccinated within the past 6 months** ^**^				0.174	0.677
No	108	760	12.49		
Yes	18	113	13.70		
**Vaccinated after the initial infection**				0.194	0.660
No	56	261	17.70		
Yes	70	299	18.97		

^*^Based on the records of the Jiangsu Province Vaccination Integrated Service Management Information System; ^**^Vaccinated with COVID-19 vaccine or not since June 1, 2022.

## 4. Discussion

As the main variant of SARS-CoV-2, Omicron could significantly reduce the neutralizing efficacy of neutralizing antibodies with different epitopes. Over 85% of the tested neutralizing antibodies were escaped by Omicron (14). The Omicron variant escaped almost all clinically approved antibody therapeutics, significantly impaired humoral immunity elicited by natural infection and vaccination, and had higher transmission rates among household contacts than those of the Delta variant, attributing to a higher risk of yet another resurgence of the pandemic (15).

COVID-19 previously infected individuals reported locally in Yangzhou city in recent 3 years were chosen. According to the weekly report of the Chinese Center for Disease Control and Prevention (CDC), during the period from December 2022 to February 2023, when COVID-19 prevention and control measures were relaxed, an estimated 82.4% of the population in China was infected (16), while the reinfection incidence of previously infected individuals in this survey was only 12.61%, far lower than that of the general population. Multiple surveys of reinfection of previously infected individuals have been conducted in different countries and regions at different periods. For example, France conducted three reinfection surveys at different periods, with reinfection incidences of 0.08% (June 2020 to February 2021), 0.4% (March 2020 to August 2021), and 3.1% (March 2021 to February 2022), respectively (17–19). 1.8% of Peruvian healthcare workers might have been reinfected with SARS-CoV-2 between March 2020 and August 2021 (20). The overall SARS-CoV-2 reinfection incidence was found to be 28.3% (95% CI: 23.7%–33.2%) in Guangdong Province between December 2022 and January 2023 (12). Chengdu reported 8.71% COVID-19 reinfection incidence during February-December, 2022 (13). The conclusions differed among countries and regions, mainly due to the different periods of the investigations and the different types of virus strains which were initially infected and reinfected. A stratified survey of individuals previously infected with different strains of the virus was conducted in the Yangzhou region. The Omicron variant reinfection incidences among previously infected individuals were 42.11% for the original strain, 19.17% for the Delta variant, and 1.00% for the Omicron variant, respectively. There were significant differences in the reinfection incidence of previously infected individuals with different strains of SARS-CoV-2. The reinfection incidence of infected individuals with the initial infection of the Omicron variant is significantly lower than that of those with the Delta variant, mainly because the immune system responded more strongly to the same type of SARS-CoV-2 strains that have been exposed again, meanwhile, produced a high level of neutralizing antibody; However, the reaction to the other newly exposed strains was weak, which can not produce neutralizing antibodies or can only produce low levels of neutralizing antibodies. This phenomenon was called original antigenic sin (OAS), also known as immune imprinting (21). A survey was conducted in the UK between February 2020 and November 2022 to investigate the reinfection with different types of SARS-CoV-2 strains among previously infected individuals, with reinfection incidences ranging from 0.3 to 16.6% (7). The results showed that there was some cross-immune protection between the different strains and that the protection gradually weakened over time. The same types of SARS-CoV-2 strains with different branches had a stronger protective effect and a lower possibility of reinfection. Of course, the individuals infected with COVID-19 during the domestic outbreak might have a stronger awareness of protection, leading to changes in health habits with limited social activities, and the data on reinfection incidence may be underestimated compared with the general population.

Reinfection referred to the reinfection of the same or different types of SARS-CoV-2 strains after the removal of the initial infection with COVID-19, due to factors such as the immune system not producing enough neutralizing antibodies after the initial infection, or the mutation of the key site of receptor-binding domain (RBD) of SARS-CoV-2 S protein and other factors. Reinfection was different from the recurrence of positive (re-positive) nucleic acid detection, which was the result of persistent/fluctuant viral shedding or sample detection problems leading to a re-positive SARS-CoV-2 nucleic acid test within a short time after the initial infection. The interval for “re-positive” was usually short, while the reinfection was long. The interval of reinfection reported among countries and regions varied greatly. The relatively long intervals of reinfection reported in this study were mainly related to the strict prevention and control policies adopted by Yangzhou City. However, the shortest intervals for reinfection reported in different regions were inconsistent either, and it was 73 days in this survey. The intervals for “re-positive” and reinfection were not entirely consistent in reports and criteria of judgment around the world. An analysis of post-discharge re-positive in Guangdong Province (22) found that up to 85.27% of re-positive cases were confirmed within 14 d after discharge. Many other regions (23–25) had also reported “re-positive” within 14d−30d, but among the re-positives, there was no fever or other symptoms and almost no secondary cases. The definition of reinfection also varied among different countries. Following the criteria established by the US CDC, SARS-CoV-2 reinfection was defined as an infection occurring more than 90 days after the collection of the first positive specimen (26); and 60 days for the French Ministry of Health (19); The UK Office for National Statistics had set multiple standards for reinfection, one of which was met to qualify as reinfection, including an interval of at least 60 days (7). Therefore, it was difficult to distinguish between reinfection and “re-positive” by the SARS-CoV-2 nucleic acid test alone. A comprehensive assessment of factors such as intervals, clinical symptoms, and epidemiologic history was required.

Additionally, our survey revealed the incidence of COVID-19 reinfection varied with different population characteristics. The risk of reinfection was significantly higher among females and younger cases, which is consistent with some other research findings (27, 28). The higher screening rate for females and younger people, and the higher exposure to occupational and social activities, might be the reasons. Medical staff and long-term care facility residents had a slightly higher reinfection incidence and were persistently at a higher risk of exposure when compared to the general population (29, 30). However, in this survey, there was no statistically significant difference in reinfection incidences among different occupations, indicating that occupational exposure in Yangzhou did not affect the reinfection incidence. Reinfection rates differed significantly among individuals initially infected with various SARS-CoV-2 strains. The reinfection incidence for those primarily exposed to the Omicron variant was 1.00%, significantly lower than that for those initially infected with the Delta variant and original strain. The timing of the emergence of dominant SARS-CoV-2 strains has been influenced by different prevention and control policies around the world, along with the definition of reinfection and various natural and social factors, all led to varying results of reinfection incidences among studies.

In this study, fewer reinfections were observed among the respondents with three doses of COVID-19 vaccination compared to the respondents with two doses or without COVID-19 vaccination, which was consistent with the conclusion of Altarawneh et al. (31). Hybrid immunity induced by a combination of natural infections and vaccinations had not been detected in providing excellent protection against SARS-CoV-2 reinfection in this survey, which may be related to factors such as the small sample size of the study population, vaccine type, and vaccination time. However, several researches demonstrated that hybrid immunity was more protective against symptomatic disease and progression to critical illness and was associated with a longer time to reinfection (32–34). Therefore, it is recommended that both first-time infected and reinfected populations should continue to strengthen vaccination efforts to reduce the incidence of reinfection or critical cases.

This study has several limitations that warrant consideration. First, there was a certain bias in the investigation process, as some of the previously infected individuals in this study were lost to interviews, and the previously infected individuals in Yangzhou had more intervened behavioral habits before the adjustment of epidemic prevention and control policies.

Second, the SARS-CoV-2 strains that infected some of the study subjects were directly identified based on the genome sequencing results of the key cases in the chain of transmission during the local epidemic. Although there were close epidemiological associations, individual abnormal situations might exist.

Third, the type of COVID-19 vaccine was not specifically analyzed as the main immunization protocol was with an inactivated vaccine. While adenovirus vector vaccine and recombinant protein (CHO cell) vaccine had relatively low vaccination rates. Additionally, as the sample size of this study was small, large-scale studies with extended follow-up periods are warranted to confirm relatively accurate conclusions.

The results of this study showed that after the epidemic period of COVID-19, there were significant differences in the reinfection incidence of previously infected individuals with different strains of SARS-CoV-2, indicating there was some cross-immune protection against various strains of SARS-CoV-2, and that the cross-immunoprotection gradually weakened over time. The infection incidence is influenced by various factors such as viral characteristics, vaccination, epidemic prevention policies, and individual variations. As the SARS-CoV-2 continues to mutate, vaccination and appropriate personal protection have practical significance in reducing the risk of reinfection.

## Data availability statement

The raw data supporting the conclusions of this article will be made available by the authors, without undue reservation.

## Ethics statement

The studies involving humans were approved by the Ethics Committee of Yangzhou Center for Disease Control and Prevention. The studies were conducted in accordance with the local legislation and institutional requirements. Written informed consent for participation in this study was provided by the participants' legal guardians/next of kin.

## Author contributions

YW: Investigation, Methodology, Software, Writing—original draft, and Writing—review and editing. JL: Formal analysis, Investigation, and Writing—review and editing. HY: Formal analysis, Investigation, Software, and Writing—review and editing. LZ: Software, Supervision, and Writing—review and editing. JH: Funding acquisition, Software, Supervision, and Writing—review and editing. LX: Data curation, Formal analysis, and Writing—review and editing. JZ: Investigation, Methodology, and Writing—review and editing. XZ: Conceptualization, Writing—review and editing, and Supervision. YH: Validation, Resources, and Writing—review and editing. YD: Investigation, Writing—review and editing, and Formal analysis. CW: Investigation, Writing—review and editing, and Formal analysis.
