# Supplementary material for: Epidemiological and clinical characteristics of COVID-19 reinfection during the epidemic period in Yangzhou city, Jiangsu province
Source: Front Public Health. 2023 Sep 13;11:1256768. doi: 10.3389/fpubh.2023.1256768 (PMC10535086; doi:10.3389/fpubh.2023.1256768)
Supplement: Supplementary file 1 [file Data_Sheet_1.docx]

**Supplementary material**

Additional information of genome sequencing: We selected samples for genome sequencing based on the availability of specimens collected during the study period and the viral load (Ct ≤32) of the RNA extracted from these specimens. Reverse transcription and multiplex PCR amplification were performed on all nucleic acid samples using the ULSEN SARS-CoV-2 genome capture kit (Since the samples were collected over a long period, we have used the sequencing kits from several manufacturers, including Hangzhou baiyitech, illumina COVID Seq, and so on), following the manufacturer’s instructions. The DNA libraries were prepared using one of the three Novel Coronavirus Library Construction Kits (NextSeq2000, CD, and illumina DNA Prep), with each sample linked to a unique barcode for sample identification. High-throughput sequencing was performed on the Illumina Iseq100 or NextSeq2000 platforms using a matched reagent kit with cycle lengths of 150.
